# Speckle-Tracking Echocardiography Elucidates the Effect of Pacing Site on Left Ventricular Synchronization in the Normal and Infarcted Rat Myocardium

**DOI:** 10.1371/journal.pone.0099191

**Published:** 2014-06-10

**Authors:** Michal Mor, Wesam Mulla, Sigal Elyagon, Hovav Gabay, Shani Dror, Yoram Etzion, Noah Liel-Cohen

**Affiliations:** 1 Cardiac Arrhythmia Research Laboratory, Department of Physiology and Cell Biology, Faculty of Health Sciences, Ben-Gurion University of the Negev, Beer-Sheva, Israel; 2 Department of Emergency Medicine, Recanati School for Community Health Professions, Faculty of Health Sciences and PREPARED Center for Emergency Response Research, Ben-Gurion University of the Negev, Beer-Sheva, Israel; 3 Division of Internal Medicine, Soroka University Medical Center, Beer-Sheva, Israel; 4 Cardiology Department, Soroka University Medical Center, Beer-Sheva, Israel; University of Minnesota, United States of America

## Abstract

**Background:**

Right ventricular (RV) pacing generates regional disparities in electrical activation and mechanical function (ventricular dyssynchrony). In contrast, left ventricular (LV) or biventricular (BIV) pacing can improve cardiac efficiency in the setting of ventricular dyssynchrony, constituting the rationale for cardiac resynchronization therapy (CRT). Animal models of ventricular dyssynchrony and CRT currently relay on large mammals which are expensive and not readily available to most researchers. We developed a methodology for double-site epicardial pacing in conscious rats. Here, following post-operative recovery, we compared the effects of various pacing modes on LV dyssynchrony in normal rats and in rats with ischemic cardiomyopathy.

**Methods:**

Two bipolar electrodes were implanted in rats as follows: Group A (n = 6) right atrial (RA) and RV sites; Group B (n = 7) RV and LV sites; Group C (n = 8) as in group B in combination with left coronary artery ligation. Electrodes were exteriorized through the back. Following post-operative recovery, two-dimensional transthoracic echocardiography was performed during pacing through the different electrodes. Segmental systolic circumferential strain (Ecc) was used to evaluate LV dyssynchrony.

**Results:**

In normal rats, RV pacing induced marked LV dyssynchrony compared to RA pacing or sinus rhythm, as measured by the standard deviation (SD) of segmental time to peak Ecc, SD of peak Ecc, and the average delay between opposing ventricular segments. LV pacing and, to a greater extend BIV pacing diminished the LV dyssynchrony compared to RV pacing. In rats with extensive MI, the effects of LV and BIV pacing were markedly attenuated, and the response of individual animals was variable.

**Conclusions:**

Rodent cardiac pacing mimics important features seen in humans. This model may be developed as a simple new tool to study the pathophysiology of ventricular dyssynchrony and CRT.

## Introduction

Dyssynchronous contraction of the left ventricle (LV) can markedly worsen the outcome of heart failure (HF) independently of traditional risk factors [1], [2]. This pathophysiology is often derived from or exacerbated by native conduction delay, such as left bundle branch block (LBBB) or artificial pacing of the right ventricle (RV) [3]–[5]. Based on QRS widening, it is estimated that at least 25–30% of patients with HF exhibit dyssynchronous LV contraction [6], [7]. Clinical studies employing biventricular (BIV) pacing (cardiac resynchronization therapy; CRT) to treat HF patients with dyssynchronous contraction have revealed significant benefits in terms of contractile performance, improved quality of life, and long-term survival [8].

Although dyssynchronous heart failure (DHF) has been extensively studied in mechanical terms and although the clinical effectiveness of CRT is remarkable, there are multiple issues that remain ill-defined at present. It is, for example, estimated that around 30% of patients do not benefit from CRT, and the clinical criteria to identify CRT non-responders remain obscure and controversial. In addition, there is variability in individual patient vulnerability to DHF progression in response to conventional RV pacing, which is difficult to predict at present [4], [9]. Only recently has the impact of dyssynchrony and BIV pacing on molecular signaling and the ionic properties of the myocardium been realized [9], [10]. Animal studies have shown that DHF is associated with regional molecular changes (collectively termed ‘molecular polarization’), including downregulation of Ca^2+^ handling proteins and connexin 43, activation of stress response kinases and increased expression of tumor necrosis factor in the lateral LV wall. Most of these changes can be reversed by CRT [11]. Additional studies identifying regional changes in ionic channels and multiple gene transcripts [12], [13] have revealed a highly complicated picture in which multiple players are involved. Therefore, comprehensive understanding of the effects of various pacing modes at the molecular level will require extensive additional scientific efforts.

One of the problems hampering progress in this field of research is the lack of suitable animal models that can be utilized for routine experimentation. Current studies almost exclusively utilize large animals (mainly dogs) and are based on the implantation of human devices. Such an experimental setting is highly demanding. In addition, the large animal models can not be manipulated genetically, an issue that is becoming increasingly critical when it is necessary to reveal molecular mechanisms. While rat and mouse models are extensively used in research on HF [14], these animal models are largely missing in research on DHF and CRT, mainly due to technical difficulties in electrode implantation. Two independent studies using M-mode echocardiography have managed to demonstrate dyssynchrony between the septum and the LV lateral wall in the murine heart [13], [15]. In addition, in the pioneering study of Bilchick et al. 2006 mice were implanted with a single pacing electrode on the RV free wall and were paced for seven days using an implanted miniature pacemaker. Using microarray analysis, this study demonstrated indications for molecular polarization as a result of prolonged RV pacing [13], [15]. Thus, it appears that rodents may mimic important features that are seen in large mammals in regard to the mechanical effect of ventricular pacing. However, to the best of our knowledge, the mechanical effect of RV pacing as compared with LV and BIV pacing has not yet been defined in rodents.

In the present study, using an implanted device that was developed for pacing and recordings in conscious rodents [16], we acutely evaluated the effect of pacing site on LV synchronization in normal and infarcted rats following post operative recovery. We hypothesized that the mechanical effects of LV pacing and BIV pacing should be favorable compared to RV pacing. Elucidation of this issue seems critical in order to clarify whether rodent models may be further developed as simple experimental tools to study the molecular biology of DHF and CRT. Utilizing speckle-tracking 2-dimensional echocardiography, a method that has been repeatedly validated in rats for segmental strain analysis over the past decade [17]–[20], we confirmed the existence of mechanical dyssynchrony when RV pacing was applied. In addition, we elucidated the electrical and mechanical effects of LV pacing and BIV pacing. Overall, our results support the notion that the effects of pacing in rodents can recapitulate important features observed in large animal models and humans.

## Methods

### Ethics statement

All animal studies reported in this article were approved by the institutional ethics committee, Faculty of Health Sciences, Ben-Gurion University of the Negev, Israel. At the end of all experiments animals were killed using IV KCl injection under deep isoflurane anesthesia.

### Implantable rat pacing device

**Figure 1:**
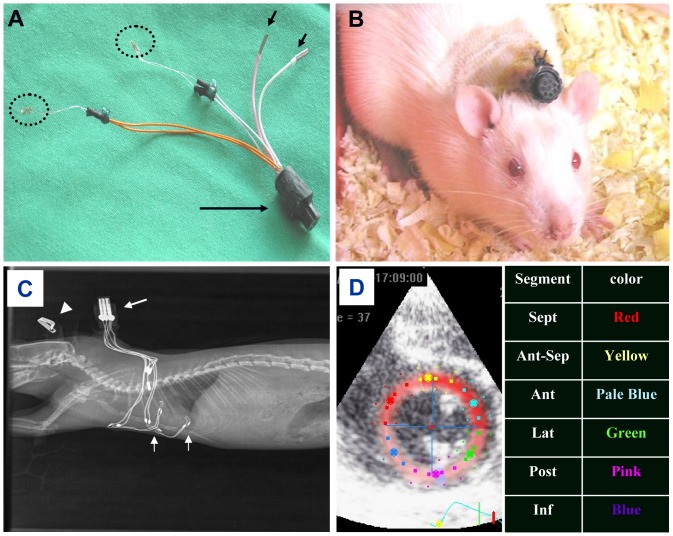
Experimental setup for LV strain analysis in paced rats. **A**: An implanted pacing device consisting of two miniature-bipolar hook electrodes (dotted circles). Long arrow; 8-pin back connector. Short arrows; peripheral electrodes for optional pseudo ECG measurements in conscious animals [16]. **B**: A rat with an exteriorized back connector following the implantation procedure. **C**: X-ray lateral view of an implanted rat. The two miniature-bipolar hook electrodes (vertical arrows at the bottom) were inserted on the RV and the LV as described in the text. The X-ray view is shown for illustration and was not utilized to access the position of the electrodes on the heart. D: Short axis mid-level echocardiographic view of an implanted rat and standard separation map of the LV into six segments for circumferential strain (Ecc) analysis.

The implantable device that was used to pace the heart at different locations was originally designed for long-term atrial pacing and recording in freely moving rats [16]. In this device, the electrical connections to the epicardial surface are achieved using miniature-bipolar hook electrodes, which have been described in detail previously [16]. The specific design of the miniature-bipolar hook electrode allows it to be positioned on a desired location on the epicardial surface of the atria or ventricles through a small lateral thoracotomy without the need of additional suturing [16]. The implantable device consists of an 8-pin ‘female’ connector that is attached by flexible insulated electrical wires to a set of two miniature bipolar hook electrodes (Figure 1a). Peripheral electrodes that are implanted on the back for pseudo ECG measurements also exist (Figure 1a,c). Before implantation, the 8-pin ‘female’ connector was covered with a latex sheath to prevent direct contact with the subcutaneous tissue during the operation. Electron beam radiation was applied for sterilization of the device before use.

### Surgical procedures

Adult male Sprague-Dawley rats (250–300 g) were anesthetized (ketamine/xylazine 75/5 mg/kg, IP) and mechanically ventilated. The animals were placed supine on a warmed heating and under sterile conditions a skin incision in the left thorax was performed and a subcutaneous tunnel was extended posteriorly to the upper back of the animal. The 8-pin ‘female’ connector (covered with a latex sheath) was inserted through the subcutaneous tunnel. Thereafter, lateral thoracotomy was performed and the two miniature -bipolar hook electrodes were positioned as follows: Group A; on the right atrium (RA) and RV apex (RV). Group B; on the RV and on the posterobasal aspect of the left ventricle (LV). Group C; similar electrode implantation as in group B, as well as proximal left anterior descending coronary artery ligation to induce myocardial infarction (MI). In order to insert electrodes on the right side (for positioning on the RA and RV) a small subcutaneous tunnel was done in the chest wall through which the electrodes were moved to the right side of the thorax. The coronary artery was ligated by 6/0 surgical suture and MI was confirmed by the presence of regional cyanosis in the myocardial surface. Following chest closure, each animal was placed in a prone position and the back connector was exteriorized through the skin of the back (Figure 1B). Postoperative recovery and analgesia were done as described previously [16].

### Cardiac pacing and echocardiograpy

Normal rats were evaluated 6–8 days following the surgical procedure. MI rats were evaluated 14 days following the surgical procedure. For the echocardiograpic analysis, each animal was sedated (isoflurane 1%) and placed on a heating pad. ECG recordings (Nihon Kodhen, RMC1100) were monitored online throughout the experiment and were also digitized and stored for offline analysis in each pacing mode (PCI-6024E, National Instruments, Austin, TX, USA). Electrical stimulation (2 ms square pulses) was applied through isolation units (Iso-Flex, AMPI, Israel) at a double diastolic threshold. The cycle length that was used to induce override pacing in the normal rats was set to 150 ms (400 bpm) which invariably induced override pacing in these animals. In the MI rats, baseline heart rate was sometimes slightly higher than 400 bpm. Therefore, the override pacing cycle length was set to 140 ms (428 bpm), which managed to override the spontaneous heart rate of these animals. During BIV pacing, two independent isolation units were used for simultaneous but independent RV apical and LV pacing. A program developed by YE [16] was used to control data acquisition, electrical stimulation and off-line analysis. QT interval measurements were manually done off-line on the digital signals under high time and voltage magnification scales. These measurements were performed using dedicated cursors which marked the isoelectric line and stressed out the time of return of the ECG signal to this line. The measured QT intervals of five independent complexes were averaged in each condition to reduce sampling error. Two-dimensional transthoracic echocardiography was performed with a Vivid 7 echocardiography system (GE Healthcare, Milwaukee WI, USA) by short-axis mid-ventricular scans at the level of the papillary muscle tips. Images were obtained with a 10 S transducer (5.4∼11.8 MHz) using an image depth of 2.5 cm and a frame rate of 225 frames/s as was repeatedly validated in the literature in rat studies [17]-[20]. The acquisition protocol was as follows: Following optimal positioning of the transducer on the chest wall, it was fixed in place by the operator, while 2D clips (20 s each) were sequentially recorded in sinus rhythm followed by override pacing at the different pacing sites. Of note, we took special care that the transducer will stay in the exact same position throughout the experiment to allow for accurate comparison between the different pacing modes in each animal. The procedure was always done by two experimentalist, one acquiring there echocardiography clips and the other controlling the pacing and confirming continuous electrical capture in each pacing mode. At the end of each experiment, the animal was sacrificed and correct positioning of the epicardial electrodes was confirmed by post mortem operation. Offline analysis of LV fractional area shortening (LV-FAS) was done by calculating the LV areas during maximal systole (LVAs) and during maximal diastole (LVAd) and then expressing LV-FAS as: (LVAd-LVAs)/LVAd×100.

### Circumferential 2-dimensional strain analysis

**Figure 2:**
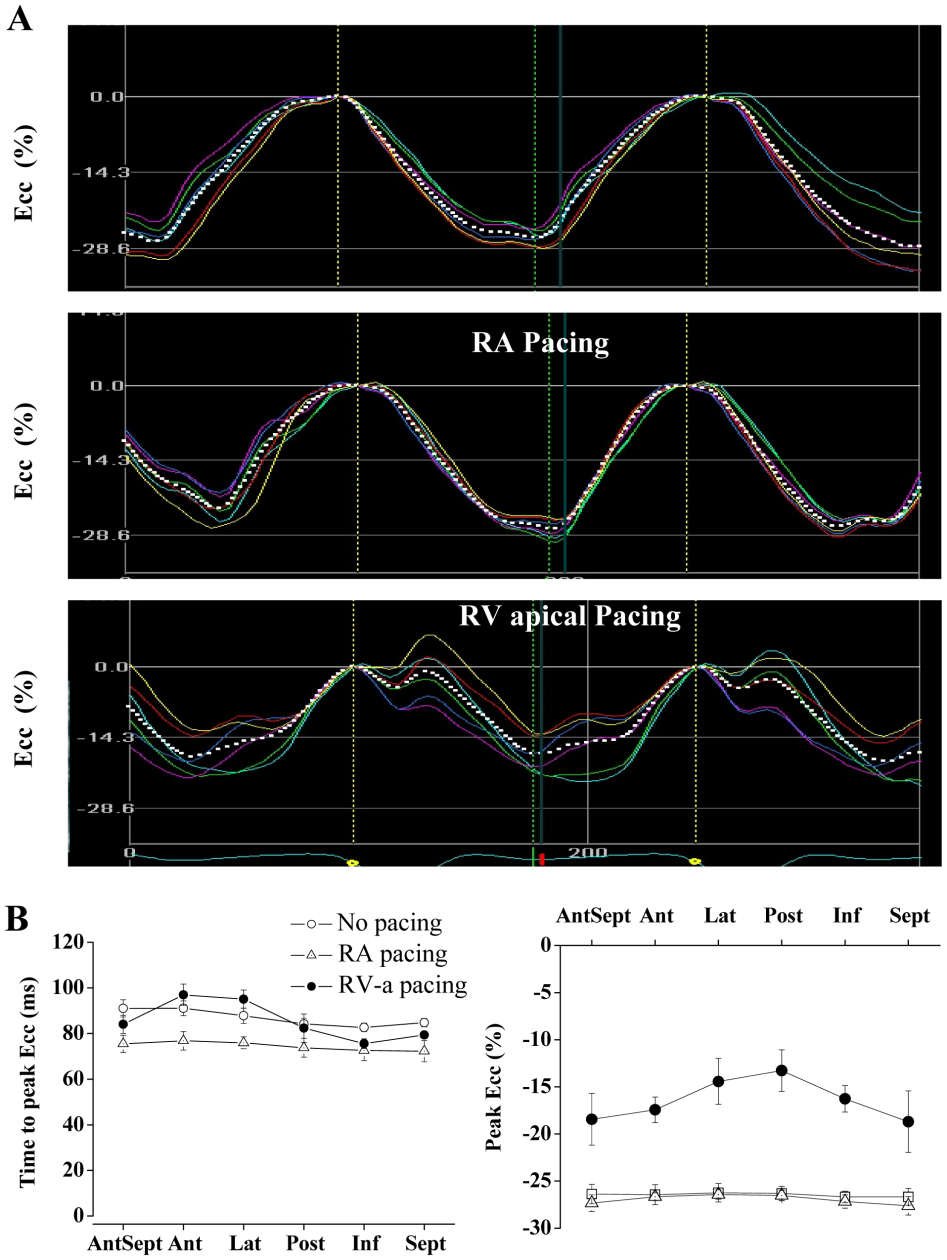
Segmental Ecc analysis during RA and RV pacing. **A**: Representative strain waveforms in an instrumented rat subjected to no pacing (upper), RA pacing (middle) and RV (lower). The colors of the different waveforms match those in the segmental map in Figure 1D. White dotted lines represent the global (average) strain of all six segments. **B**: Average segmental time to peak Ecc (left) and peak Ecc (right) in seven animals subjected no pacing, RA pacing, and RV pacing.

Based on the existing literature in rodents, we restricted our current analysis to circumferential strain (Ecc) which is more reliable than radial strain in the small heart of rats [18], [19]. Ecc was evaluated as described in the literature [17]–[19]. Briefly, analysis of mid-level short-axis images was done with dedicated software (“2D-strain”, EchoPAC, GE healthcare, Vingmed, Norway). Endocardial border at end-systole and the region of interest from the endocardium to the epicardial edge were determined by the user (Figure 1d). Thereafter, an automatic speckle tracking analysis was performed for the short-axis images to track the motion of speckles contained within the region of interest on a frame-to-frame basis [21]. Ecc curves, expressed in percentage values as a function of time were exported to a customized program (MATLAB software, MathWorks) to calculate the time from onset of QRS to peak Ecc (time to peak Ecc) and the value of maximal Ecc (across the six mid-level segments (Figure 1d, Figure 2a). The values of maximal Ecc are shown as % of segmental length at onset QRS complex. At least five consecutive beats were analyzed under each pacing condition in each animal. Initial determination of the region of interest from the endocardium to the epicardial edge was performed under no pacing conditions and was kept as constant as possible during the different pacing conditions. Analysis of all rats in each group was done by a single observer (MM - normal rats, WM - MI rats). An independent observer who is highly experienced in echocardiographic methodologies including 2D strain analysis (NLC) confirmed the consistency of the analysis performed by the two observers.

### Statistical analysis

Values are expressed as means ±SE. Due to the relatively small sample size in each group, analysis of the difference between pacing conditions was done using Kruskal-Wallis one way analysis of variance on ranks with Student-Neuman-Keuls post hoc test. All statistical tests were done using SigmaStat 3.1 (Systat Software, Inc, Point Richmond, CA). The criterion for significance was set at *P*<0.05.

## Results

### RV pacing versus RA pacing or sinus rhythm in normal rats

**Figure 3:**
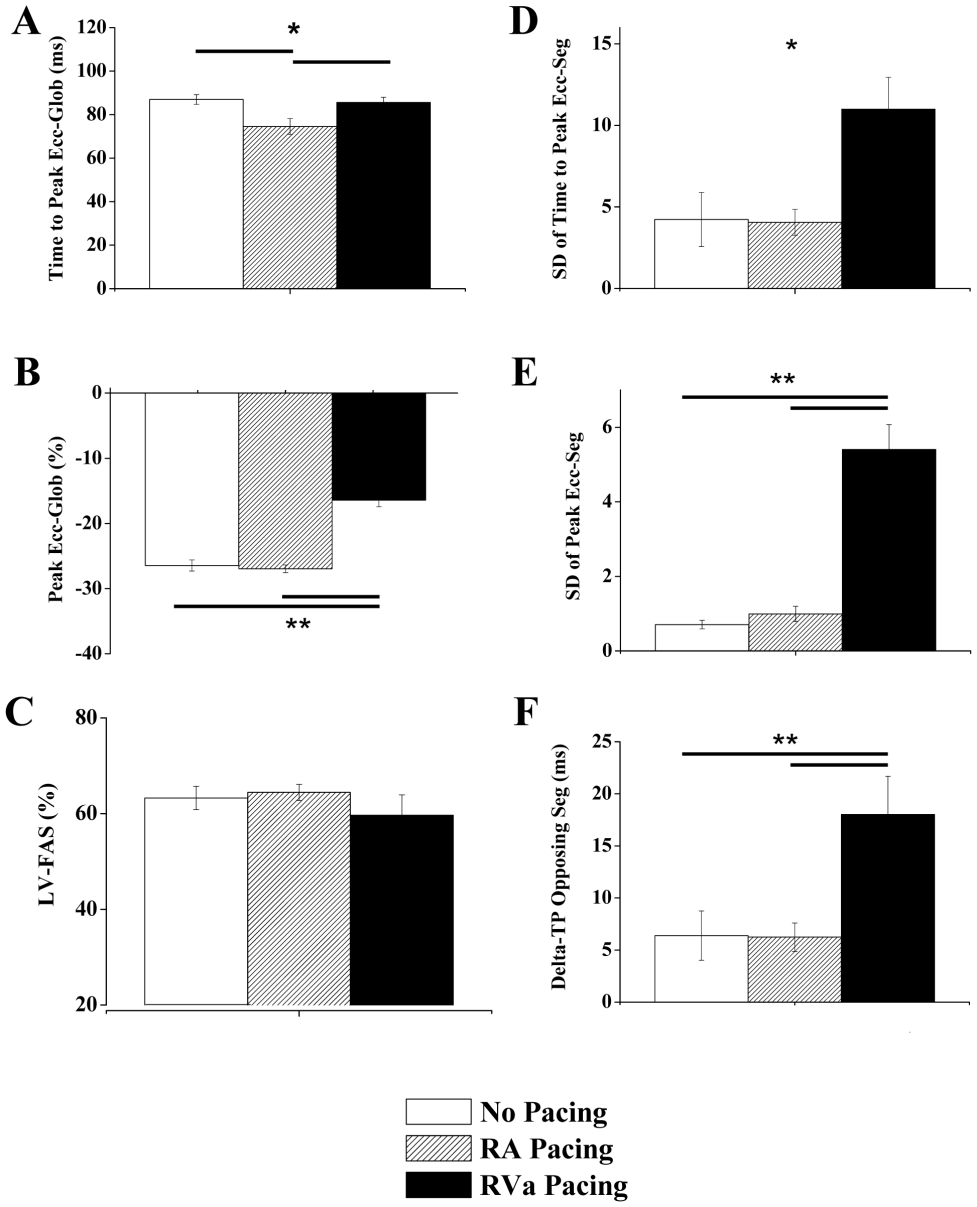
RV pacing induces marked LV dyssynchrony compared to RA pacing. Summary of echocardiographic parameters in group A subjected to no pacing, RA pacing, and RV pacing. **A**: Average time to peak Ecc of all six segments (global). **B**: Average peak Ecc of all six segments (global). **C**: LV fractional area shortening. **D**: Standard deviation of the time to peak Ecc of different segments. **E**: Standard deviation of the peak Ecc of different segments. **F**: Analysis of ‘delta TP opposing segments’, i.e., the average time difference between the peak Ecc of opposing segments. * P<0.05, ** P<0.01. Horizontal lines above\below the bar graphs indicate significant differences between pacing modes in the post-hoc analysis. Note that in **D** the Kruskal-Wallis test was significant. However, the post-hoc analysis could not reveal the source of difference between the groups.

As a first step we aimed to compare the effect of RV pacing to both no pacing and RA pacing modes. This analysis was done in order to confirm that the override pacing itself does not induce rate-dependent LV dyssynchrony compared to the no pacing mode (sinus rhythm). For that purpose, group A (n = 6) was instrumented with a bipolar RA pacing electrode and a bipolar RV pacing electrode, and override pacing in these two locations (150 ms CL) was compared to the no pacing state. The obtained Ecc traces of the different LV segments typically demonstrated no major differences between RA pacing and sinus rhythm. In contrast, RV pacing invariably induced marked changes in the segmental strain pattern. The RV pacing typically produced an early systolic contraction in the inferior or posterior segments, which was associated, in some cases, with a clear stretching effect in opposing segments (Figure 2A). Summary of the segmental time to peak Ecc and the segmental peak Ecc patterns under the different pacing modes is presented figure 2B. Analysis of important global parameters derived from the segmental Ecc analysis under the different pacing modes is shown in Figure 3. The global time to peak Ecc was found to be slightly shorter during RA pacing compared to no pacing (85.6±4.1%, p<0.05) an was also shorter compared to RV pacing (Figure 3A). Global peak Ecc (Figure 3B) was not different between RA pacing and no pacing but was markedly attenuated during RV pacing (61.0±3.7% compared to no pacing, p<0.01 compared to both RA pacing and no pacing). While the Ecc analysis identified a marked effect of RV pacing on strain parameters, concurrent analysis of LV-FAS did not identify a significant effect of the different pacing modes on this parameter (Figure 3C, see discussion). Specific analysis of LV dyssynchrony variables indicated marked deleterious effects of RV pacing on all the calculated parameters, including standard deviation of time to peak Ecc (Figure 3D), standard deviation of peak Ecc (Figure 3E) and the average time difference between the peak Ecc of opposing segments (Delta TP opposing segments, Figure 3F). There was no statistical difference in these parameters between RA pacing and sinus rhythm.

### Effects of different ventricular pacing modes in normal rats

**Figure 4:**
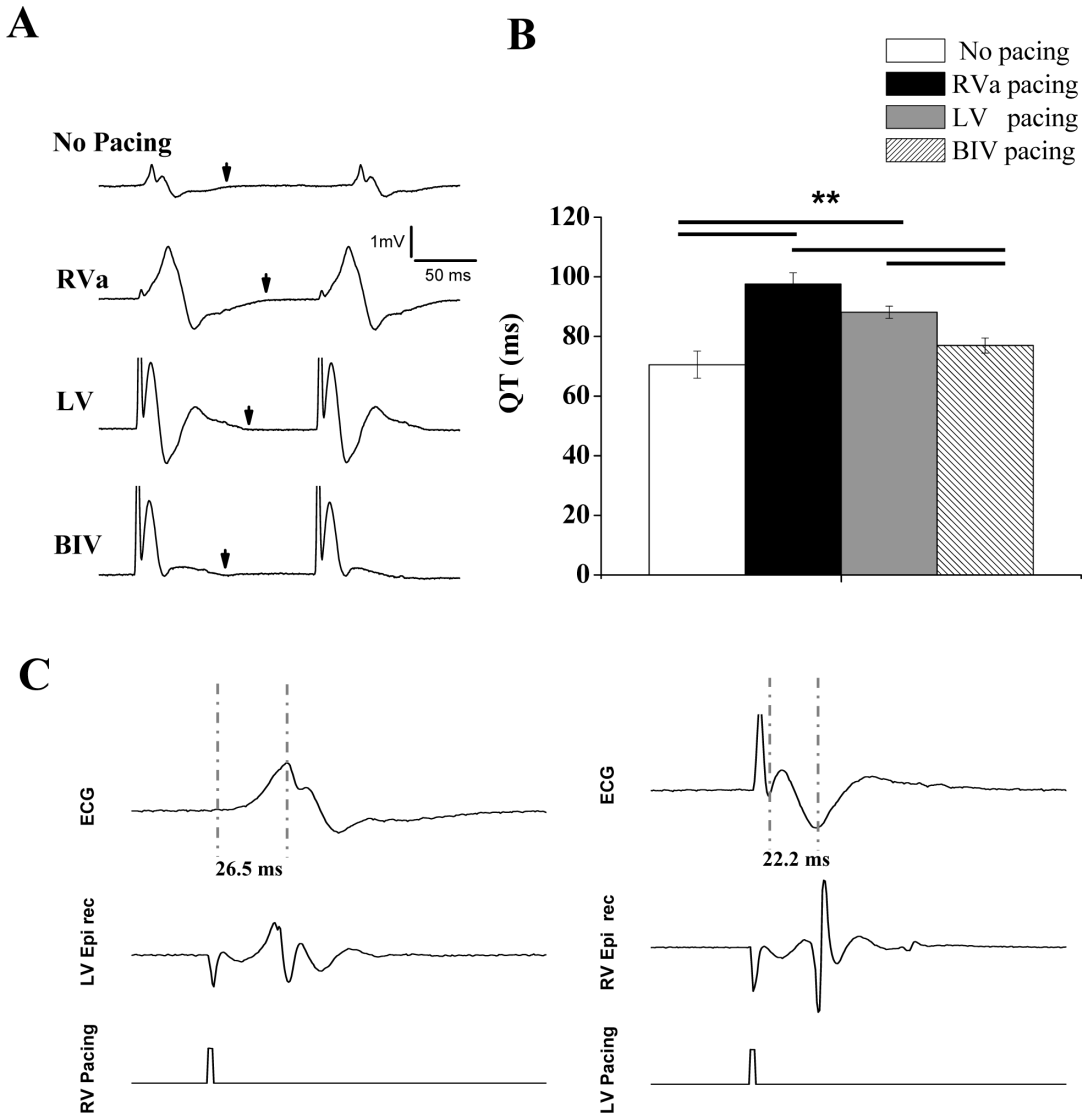
RV pacing induces electrical dyssynchrony compared to LV and BIV pacing. **A**: Representative ECG recordings from an instrumented rat during sinus rhythm (no pacing) and during RV, LV and BIV pacing. Arrows indicate the termination of the T wave in each trace. **B**: Summary of QT interval analysis in six instrumented rats. ** P<0.01. Horizontal lines above the bar graph indicate significant differences between pacing modes in the post-hoc analysis. **C**: Example of epicardial electrograms during RV pacing (left) and LV pacing (right). In each recording upper trace is a peripheral ECG signal (Lead I), middle trace is the epicardial electrogram and lower trace shows the time of cardiac pacing. Note that the epicardial activation in the RV electrode correlates with a late negative vector in the ECG signal. In contrast, the epicardial activation during RV pacing is correlated with a late positive vector in the ECG signal. Measurements of the time from end of stimulus artifact to the peaks of these late vectors in the ECG (dash vertical lines) indicate shorter activation time during LV pacing. Analysis of epicardial electrograms in an additional animal from group B demonstrated similar findings (not shown).

In the next stage, we evaluated group B (n = 7) rats, which had bipolar pacing leads in the RV and LV. The effect of override pacing in these two locations alone or simultaneously (BIV pacing) was compared to the no pacing mode. In terms of electrical activity, the ECG recording indicated QRS prolongation during RV pacing as compared to LV and BIV pacing (Figure 4A). However, the unclear separation between the QRS and the T-wave in the rodent heart [22], [23], did not allow quantitative analysis of QRS width during the various pacing modes. Nevertheless, an analysis of QT duration indicated marked QT prolongation during RV pacing (138.3±5.3%, p<0.01) as compared to the no pacing mode. This marked QT prolongation was reduced by LV pacing (124.9±2.9%) and even further reduced by BIV pacing (109.1±3.6%), as shown with detailed statistical analysis in Figure 4B. In addition, recordings of epicardial electrograms from the RV electrode when pacing the LV and vice versa, was performed in two animals from group B. These recordings supported a longer sequence of activation during RV pacing compared to LV pacing (Figure 4C).

**Figure 5:**
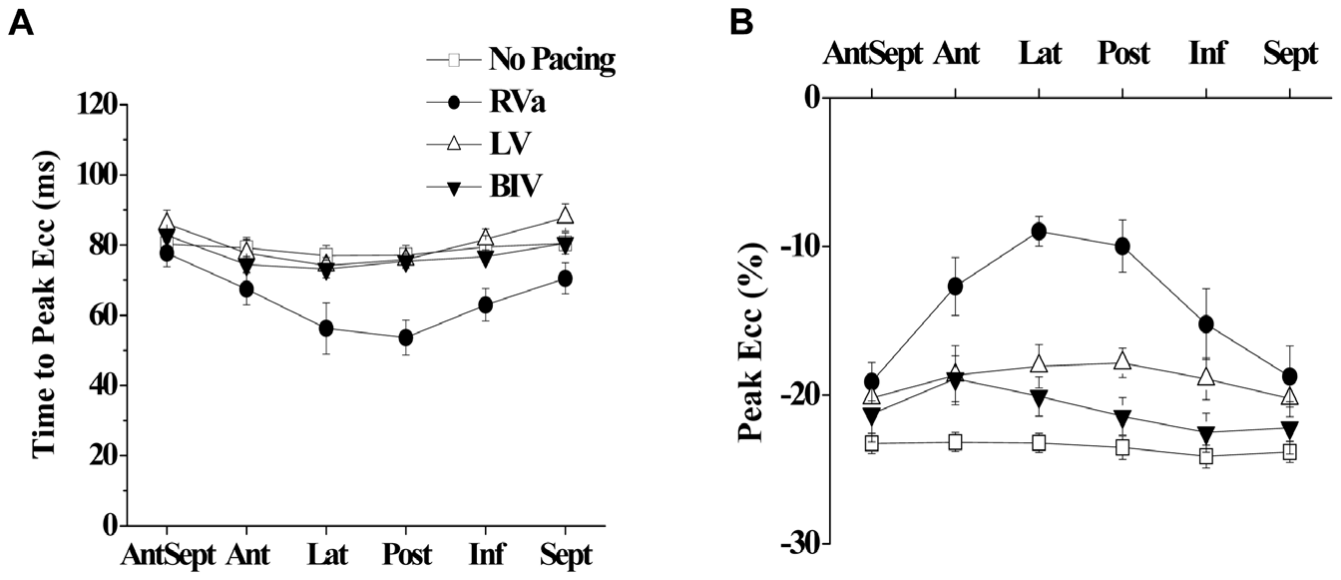
Segmental Ecc analysis during RV, LV and BIV pacing. Average segmental time to peak Ecc (**A**) and peak Ecc (**B**) in seven animals from group B subjected to no pacing, RV pacing, LV pacing and BIV pacing.

**Figure 6:**
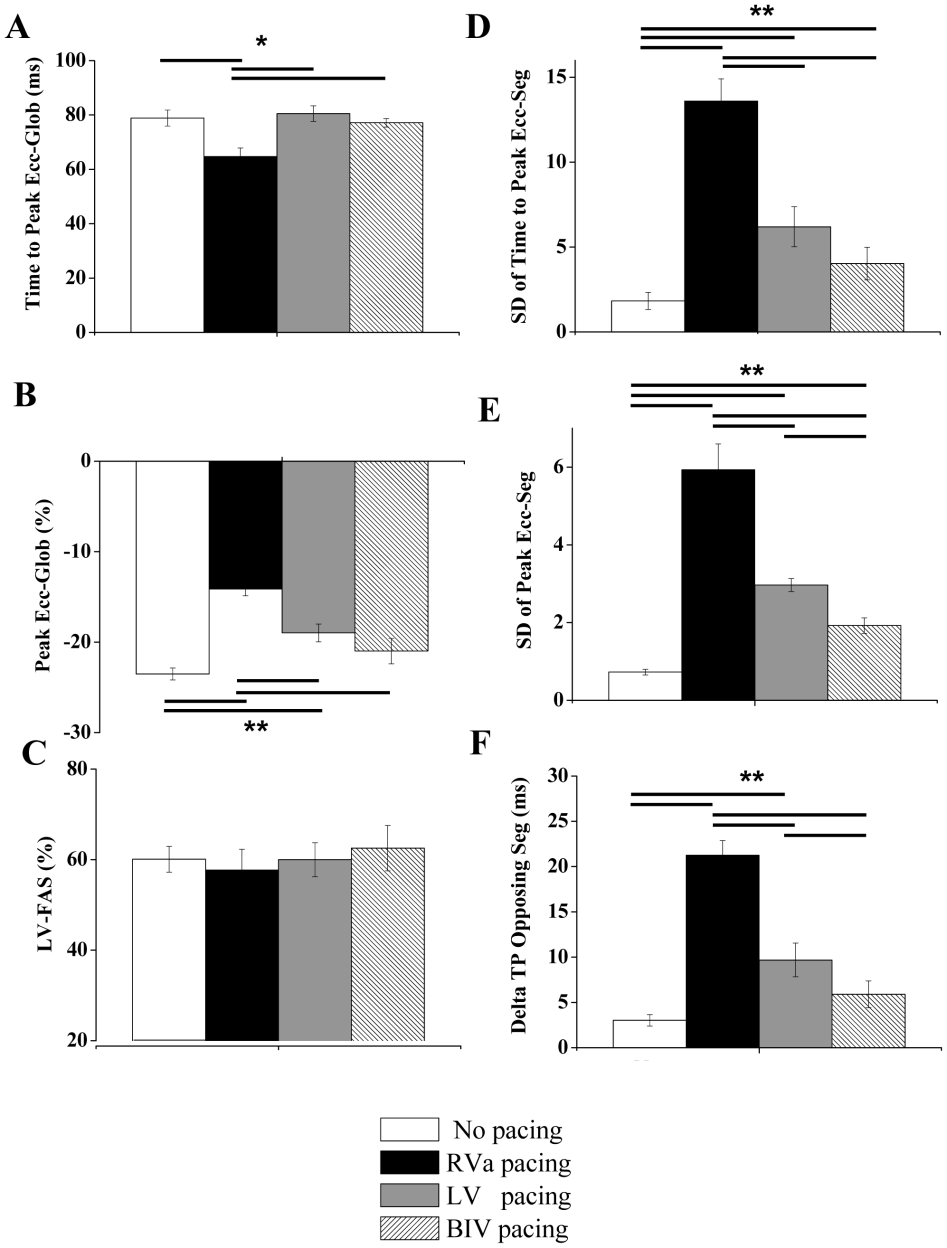
RV pacing induces mechanical dyssynchrony compared to LV and BIV pacing. Summary of echocardiographic parameters in six animals subjected to no pacing, RV, LV and BIV pacing. **A**: Average time to peak Ecc of all six segments (global). **B**: Average peak Ecc of all six segments (global) **C**: LV fractional area shortening **D**: Standard deviation of the time to peak Ecc of different segments. **E**: Standard deviation of the peak Ecc of different segments. **F**: Average time difference between the peak Ecc of opposing segments (‘delta TP opposing segments’, see Figure 3 for details). * P<0.05, ** P<0.01. Horizontal lines above\below the bar graphs indicate significant differences between pacing modes in the post-hoc analysis.

We next evaluated the mechanical dyssynchrony during the various pacing modes. Summary of the segmental time to peak Ecc and the segmental peak Ecc under the different pacing modes in group B are presented figure 5. Analysis of important global parameters derived from the segmental Ecc analysis under the different pacing modes is shown in Figure 6. RV pacing significantly reduced both the global time to peak Ecc and the global peak Ecc as compared to no pacing. These parameters recovered significantly during LV and BIV pacing (Figure 6A, B). In addition, while a prominent increase in all the parameters of LV dyssynchrony was noted during RV pacing, LV pacing and particularly BIV pacing markedly improved all three parameters of LV dyssynchrony (Figure 6 D–E). Of note, the segmental pattern of the time to peak Ecc during RV pacing (Figure 5) was somewhat different from that observed in group A (Figure 2), possibly due to variability in the positioning of the RV electrode on the epicardial surface of the RV (see limitations section in our discussion). As in group A (Figure 3), we could not detect significant changes in LV-FAS during the different pacing modes (Figure 6C).

### Effects of different ventricular pacing modes in rats with ischemic-induced heart failure

**Figure 7:**
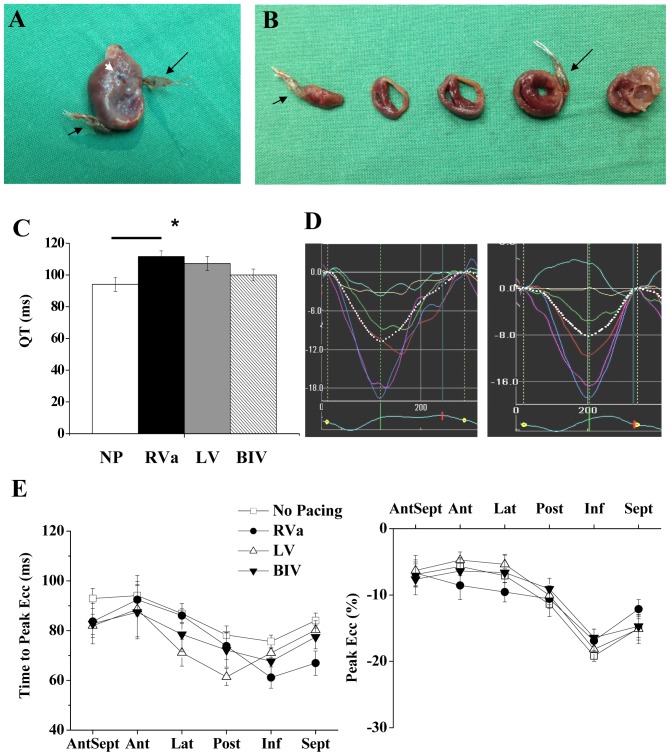
Combined model of pacing and ischemic heart failure. **A**: Anterior image of a heart subjected to electrodes implantation in combination with left coronary ligation. RV electrode and LV electrode are marked by short and long black arrows, respectively. White arrow; coronary artery ligation site. **B**: Transverse sections of the heart shown in A. Apical to basal sections are ordered from left to right. **C**: Summary of QT interval analysis in five instrumented rats with MI. * P<0.01. Horizontal line above the bar graph indicate significant difference in the post-hoc analysis. **D**: Representative strain waveforms of two instrumented rats with MI under control conditions. **E**: Average segmental time to peak Ecc (left) and peak Ecc (right) in five rats with ischemic heart failure subjected to no pacing, RV, LV and BIV pacing.

To elucidate the influence of ischemic HF on the electromechanical results of our model, group C (n = 8) was subjected to a combined procedure of electrode implantation and left coronary artery ligation. We analyzed the effect of pacing two weeks following surgery, at a time when prominent scar tissue and myocardial remodeling were present (Figure 7A, B). Out of the eight animals with confirmed MI, one had electrical capture only in the LV and was excluded from analysis. Two additional animals had electrical capture only in the RV and were therefore analyzed only in the no pacing and RV pacing modes. Final statistical analysis was done only on the five MI rats that exhibited electrical capture in both RV and LV sites. In electrophysiological terms, the MI rats demonstrated baseline prolongation of the QT interval compared to the normal rats (94.0±4.4 ms vs. 70.5±4.4 ms, p<0.001). However, the effect of pacing on the QT interval followed a similar trend to that in the normal rats. Indeed, RV pacing statistically increased the QT interval as compared with no pacing mode (118.6±3.7%, p<0.05), while the QT during LV and BIV pacing did not differ from sinus rhythm (113.9±4.7% and 106.2±3.9%, respectively, see details in Figure 7C).

**Figure 8:**
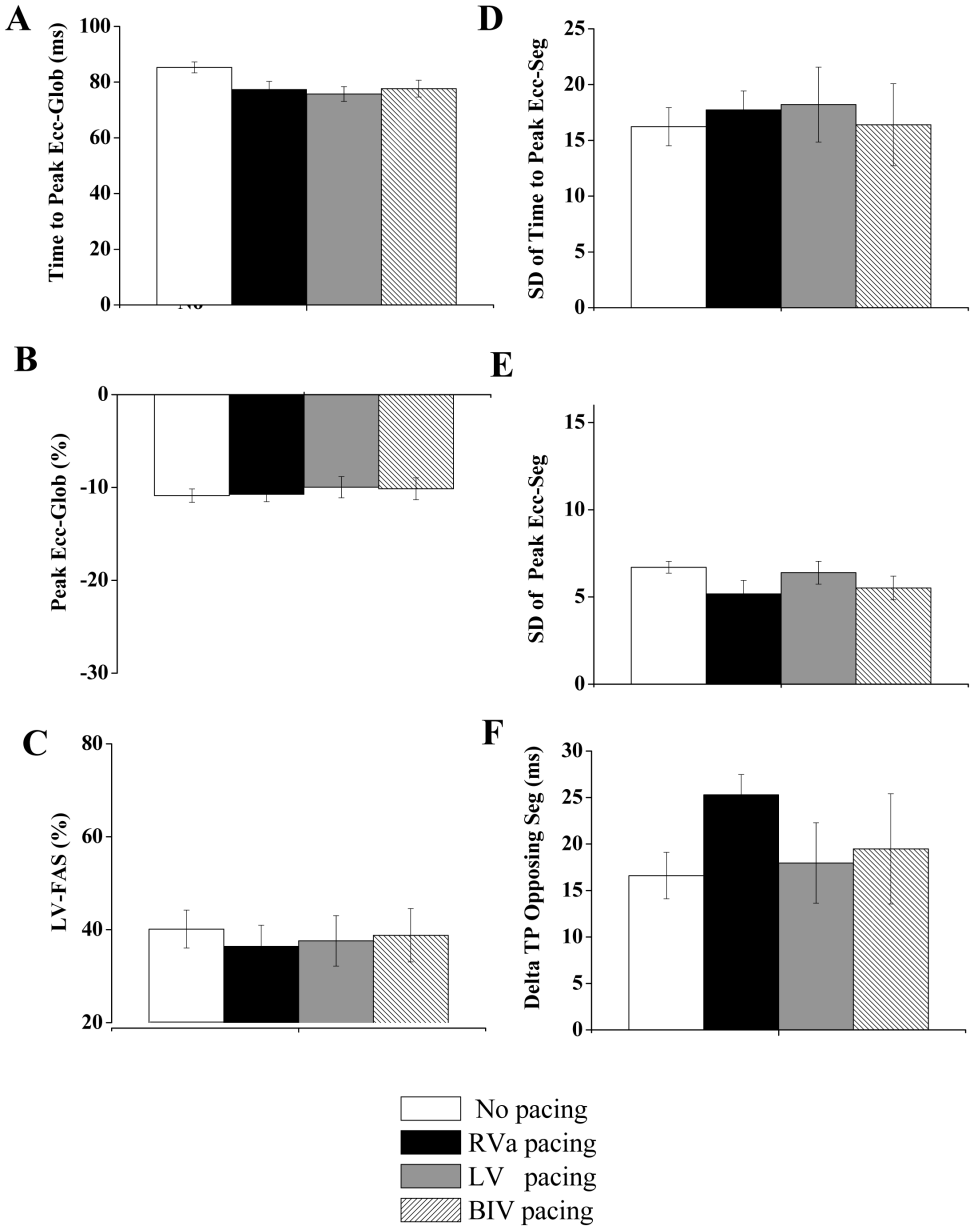
Attenuated effects of pacing in rats subjected to extensive MI. Summary of echocardiographic parameters in five rats with ischemic heart failure subjected to no pacing, RV pacing, LV pacing, and BIV pacing. **A**: Average time to peak Ecc of all six segments (global). **B**: Average peak Ecc of all six segments (global) **C**: LV fractional area shortening **D**: Standard deviation of the time to peak Ecc of different segments. **E**: Standard deviation of the peak Ecc of different segments. **F**: Average time difference between the peak Ecc of opposing segments (‘delta TP opposing segments’, see Figure 3 for details). Kruskal-Wallis test did not reveal a significant difference between the different pacing modes for any of the presented parameters.

**Figure 9:**
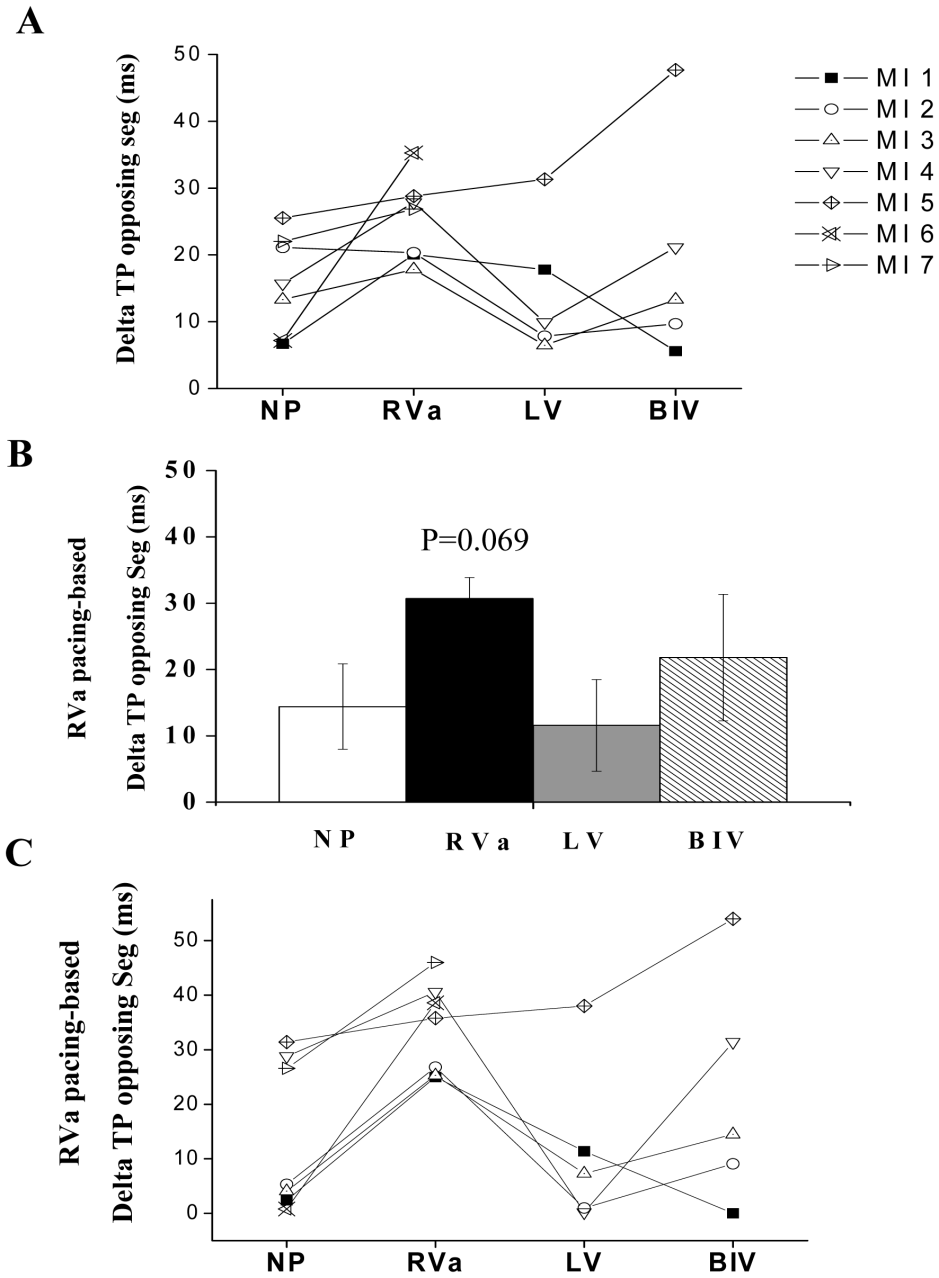
Individual variability in the effect of pacing in rats subjected to extensive MI. **A**: Average time difference between the peak Ecc of opposing segments in seven individual rats with MI (MI1 to MI7). Note the tendency of increased delta TP in all rats during RV pacing. **B**: Analysis of delta TP in each animal focused solely on the two opposing segments presenting maximal dyssynchrony during RV pacing. **C**: Delta TP focused on the two opposing segment presenting maximal dyssynchrony in seven individual rats with MI (as in Figure 9B).

Evaluation of segmental Ecc in group C revealed typical akinesia or dyskinesia in the anteroseptal, anterior and lateral segments at baseline (Figure 7D). No apparent effect of pacing on the magnitude and temporal pattern of the peak Ecc was observed in these rats (Figure 7E, Figure 8 A,B). In addition, while dyssynchrony parameters were much higher in the MI animals at baseline, there were no apparent effects of the different pacing modes on these parameters, excluding a non-significant tendency to increase in the ‘delta TP opposing segments’ parameter during RV pacing (Figure 8 D–F). Looking more closely at the effect of pacing on the ‘delta TP of opposing segments’ in individual rats (Figure 9 A), we observed a consistent tendency for this parameter to increase during RV pacing in 6 out of the 7 evaluated animals. Compared with RV pacing the effects of LV pacing and BIV pacing on the delta TP of opposing segments were variable. While these pacing modes seemed to have a beneficial effect in some cases (e.g., animals 2 and 3), there was a worsening of this parameter in animal 5 when LV and BIV pacing were applied (Figure 9 A). An additional analysis specifically focusing on the two opposing segments that had maximal temporal dyssynchrony during RV pacing detected a rather similar pattern (Figure 9 B, C).

## Discussion

The present study introduces a new model of ventricular pacing in an instrumented rat, which allowed us for the first time to systematically evaluate the effects of pacing site on LV synchronization in the rodent heart. We used speckle-tracking echocardiography analysis of Ecc, which has been reported to reliably and sensitively detect changes in LV dyssynchrony under various clinically relevant conditions in the rat heart [17]–[20]. The speckle-tracking echocardiographic methodology has previously been applied extensively in large animal models and in people and has been shown to be much more sensitive to changes in LV dyssynchrony than global echocardiographic measures, such as fractional shortening [24]. Therefore, in the present study, it is not surprising that there were no significant effects of pacing on LV-FAS, while the effects of pacing site on segmental and global Ecc pattern were consistent and significant.

The main findings of our Ecc analysis indicate that while the rodent heart has some clear differences compared to the human heart in aspects such as action potential repolarization and calcium handling [22], [23], [25], its mechanical response to the effect of various pacing modes seems to have important similarities to the large mammalian heart. While previous studies in the mouse heart have already demonstrated negative effects of acute RV pacing on LV synchrony using M-mode echocardiography, [13], [15] our study is the first to utilize speckled tracking analysis under various pacing modes in order to view the full segmental pattern during RA, RV, LV and BIV pacing. The results show clear beneficial effects of LV and to a greater extend BIV pacing on LV synchrony compared to RV pacing. In terms of electrical synchrony, we found it difficult to reliably measure the QRS duration during the different pacing modes due to the absence of apparent differentiation between the QRS complex and the T wave in the rat ECG. However, the effects of different pacing modes on the QT duration, which were consistent and significant (Figure 4 A,B), seem to reflect, at least partially, changes in QRS duration that are embedded in the QT interval. In addition, analysis of epicardial electrograms also supports longer activation time during RV pacing compared to LV pacing (Figure 4 C). Thus, LV and BIV pacing appear to induce beneficial effects on electrical synchrony compared with RV pacing in the rat heart. In terms of mechanical synchrony our results demonstrate that LV-only pacing has favorable outcome which is almost similar to the effect of BIV pacing. While many studies support the notion that LV-only pacing may have beneficial effects in HF patients [26], recent data in children with normal cardiac function and complete AV block demonstrated striking similarity to the results of our rat model in regard to LV-only pacing [27]. Moreover, LV-only pacing appears to be associated with good clinical outcome in this and other studies in the pediatric population [28], [29]. It should be mentioned that the segmental findings of our study as well as the pediatric studies stated above appear to contrast the findings of [30], which demonstrated reduced Ecc near the LV pacing site compare to remote sites near the RV in the dog model of acute pacing. However, the segmental Ecc analysis in the above study was done by MRI using a much higher spatial resolution dividing the mid LV into 24 segments and 8 longitudinal segments. Thus the analysis focused on segments which are not in the same transverse plane and are much more refined to the actual vicinity of the pacing electrodes.

The addition of MI-induced HF to the double site ventricular pacing model was performed as a proof of concept for the usefulness of our rat model in simulating a scenario that is clinically relevant. Our results indicate decreased beneficial effect of LV or BIV pacing in animals with extensive MI. These results are consistent with various clinical results indicating that the beneficial effect of CRT is highly dependent on the scar burden and is lost when the scar burden passes a certain limit [31], [32]. A recent study in a dog model indicated, however, a clear beneficial effect of CRT in the setting of ∼20% scar burden [33]. In this context, a limitation of our current analysis was the lack of a quantitative measurement of scar burden. However, based on the echocardiographic analysis of akinetic/dyskinetic segments, LV-FAS analysis (Figure 8C), and the macroscopic view of the MI in the hearts (Figure 7A, B), it appears that the MIs of the present study were rather extensive in nature and were far beyond the 20% scar burden that was calculated in the dog model of Rademakers et al. [33]. Importantly, our initial analysis did not detect a marked deleterious effect of RV pacing in the MI animals when LV dyssynchrony parameters were compared to the no pacing mode (Figure 8). This phenomenon may be partially attributed to the nature the dyssynchrony parameters that were selected in our study, which may lose their sensitivity when high values are already present at the baseline. Nevertheless, it may also be possible that in the presence of an extensive MI, the effect of RV pacing on LV dyssynchrony is indeed small due to the high LV dyssynchrony that is already present at baseline. Looking specifically at the temporal synchrony of opposing segments, which appears to be specifically useful both clinically and experimentally [19], [34], the picture that we obtained was somewhat different. Indeed, using this measure, we could identify a tendency of increased dyssynchrony during RV pacing in the majority of rats (Figure 9), a finding suggesting that this pacing still imposes a deleterious effect in the extensive MI setting but is harder to detect and analyze in this context. An examination of opposing segments also revealed marked variability in the response of individual rats with ischemic HF to the effect of LV and BIV pacing. While, in some animals, these modes of pacing seemed to be favorable compared to RV pacing, an opposite tendency was clearly noted in one out of the five fully analyzed rats. Clearly, more extensive work is required to further define the parameters that are responsible for the observed variability in the setting of MI. Quantitative analysis of scar burden and precise post mortem analysis of the location of LV pacing site in relation to the scar tissue, as well as invasive hemodynamic measurements during the different pacing modes, would all make a valuable contribution to further analysis in this complex setting.

The current rat model opens a window of opportunity for various studies regarding DHF and CRT using a rather simple and economically efficient model. The study might also pave the way to the application of double site pacing in genetically engineered mice models. New technical advances in the development of wireless pacing for mice, [35] imply that such studies may indeed become more feasible in the near future.

### Limitations

There are several limitations to the present model. The absence of atrial sensing electrode and dependence on override pacing prevents atrioventricular synchrony. However, since the main comparisons of different ventricular pacing modes all rely on override pacing, this confounder should be equal in the different ventricular pacing modes. In addition, the pseudo LBBB pattern induced by RV pacing may not necessarily represent all the features of true LBBB. In this context, the epicardial location of our RV electrodes is also important to note, since this setting differs from the most common clinical RV pacing situation, where the RV lead is endocardial. Finally, the patterns of LV dyssynchrony observed in different animals are not completely identical, as can be appreciated by comparison of the segmental time to peak patterns in Figure 2 (group A) and Figure 5 (group B). The source of this variability between the groups was not clear to us. One of the things we suspect is that although the position of electrode implantation seems identical to the operator, the small size of the rat heart did not allow for detection of subtle differences in the position of the implantation, specifically in the anterior-posterior dimension. Such difference may affect the electrical activation sequence. Thus, if an electrode is positioned in a more anterior position close to the apex, the first LV segment that is activated is the inferior one as seen in the summary of animals in group A (Figure 2C). On the other hand, if the electrode is positioned in a more posterior location then the first LV segment that is activated is the posterior one as was dominantly observed in the animals of group B (Figure 5). This possibility implies that more attention to the exact location of the RV electrode should be used in future studies. Nevertheless, our current analysis also indicates that regardless of the exact segmental pattern of the time to peak strain, the variability of this parameter is remarkably larger during RV pacing compared to LV pacing and BIV pacing.

## Conclusions

The present study shows the feasibility of an electrodes implantation procedure for double site epicardial pacing in rats, and the ability to combine this technique with conventional MI surgery. By the use of ECG recordings and speckle-tracking echocardiography we found that rodent pacing mimics important electromechanical features seen in large mammals and in people. Thus, this model may become a simple new tool to study the pathophysiology of ventricular dyssynchrony and CRT.

## Supporting Information

Checklist S1 NC3Rs ARRIVE Guidelines Checklist.
(DOC)
